# Implication of taxonomic abundance of gut microbiota in prediabetes: a systematic review

**DOI:** 10.3389/fnut.2025.1577528

**Published:** 2025-04-16

**Authors:** Iche Andriyani Liberty, Laily Hanifah, Hanna Farida Rachmat, Aidi Alifia Putri, Dessy Pratiwi, Andri Pramesyanti Pramono, Fachmi Idris, Suryadi Tjekyan

**Affiliations:** ^1^Department of Public Health and Community Medicine, Faculty of Medicine, Universitas Sriwijaya, Palembang, Indonesia; ^2^Department of Public Health Sciences, Faculty of Public Health, Universitas Sriwijaya, Palembang, Indonesia; ^3^Faculty of Health Science, Universitas Pembangunan Nasional Veteran Jakarta, Jakarta, Indonesia; ^4^Faculty of Medicine, Universitas Indonesia, Jakarta, Indonesia; ^5^Indonesia Health Development Center, Jakarta, Indonesia; ^6^Research Center for Molecular Biology Eijkman, National Research and Innovation Agency (BRIN), Cibinong, Indonesia

**Keywords:** prediabetes, gut microbiota, gastrointestinal microbiota, dysbiosis, insulin resistance

## Abstract

**Background:**

Prediabetes is defined by blood glucose levels that are higher than normal but below the diagnostic threshold for diabetes. Environmental factors associated with diabetes may contribute to its development through alterations in the gut microbiota. Recent studies suggest that changes in the composition and function of the gut microbiota play a role in the pathogenesis of diabetes mellitus and other metabolic disorders.

**Objective:**

This study aims to systematically examine taxonomic abundance and its implications in the gut microbiota of individuals with prediabetes, identify key dysbiotic patterns, and explore their potential role in inflammation, insulin resistance, and progression to type 2 diabetes.

**Methods:**

We conducted a systematic literature review following PRISMA guidelines. The review included sources from PubMed, ClinicalKey, ScienceDirect, Springer, and Scopus. We retrieved original research articles published in English that focused on prediabetes and gut microbiota from 2015 to the date of our search. Out of 827 full-text articles screened, 6 were selected based on defined inclusion and exclusion criteria.

**Results:**

Dysbiosis of the gut microbiota in prediabetes is characterized by a reduction in butyrate-producing bacteria such as *Faecalibacterium prausnitzii* and *Roseburia*, along with an increase in potentially harmful taxa such as *Escherichia/Shigella* and *Prevotella* species. This imbalance is associated with systemic inflammation and insulin resistance, evidenced by elevated levels of C-reactive protein (CRP), interleukin-6 (IL-6), tumor necrosis factor-alpha (TNF-*α*), and lipopolysaccharide-binding protein (LBP). Increased intestinal permeability facilitates the translocation of bacterial components such as lipopolysaccharides (LPS), further linking gut microbiota changes to the development of insulin resistance and type 2 diabetes.

**Conclusion:**

This review highlights the need for further research to explore the potential therapeutic role of gut microbiota in prediabetes.

**Systematic Review Registration:**

Prospero; Identifier CRD42025637369.

## Introduction

The human gastrointestinal (GI) tract is one of the largest interfaces (250–400 m^2^) between the host, environmental factors, and antigens in the human body. Over an average lifetime, approximately 60 tonnes of food pass through the digestive tract, introducing an abundance of microorganisms from the environment that can impact gut integrity (1). Gastrointestinal health in childhood significantly influences immune function and metabolic control, laying the foundation for long-term health outcomes. Gut microbiota and gut function during infancy and early childhood have been associated with improved immune responses, a lower risk of chronic diseases, and better overall wellbeing in adulthood (2). The assemblages of bacteria, archaea, and eukaryotes that colonize the digestive tract are collectively referred to as “gut microbiota.” These microbes have co-evolved with their hosts over thousands of years, forming complex and mutually beneficial relationships (3, 4). The number of microorganisms inhabiting the GI tract has been estimated to exceed 10^14^, outnumbering human cells by a factor of ten and containing over 100 times the genomic content (microbiome) of the human genome (3, 5). However, recent estimates suggest that the human cell-to-bacterial ratio is closer to 1:1 (6). Due to the significant number of bacterial cells in the body, the host and its microbiota are often considered a “superorganism” (5, 7). Prediabetes is characterized by blood glucose levels that are higher than normal but below the diagnostic threshold for diabetes. Individuals with prediabetes frequently present with overweight and insulin resistance (8).

Insulin resistance is the primary underlying mechanism of metabolic syndrome, diabetes, and prediabetes. It is also a key factor in obesity-related cardiometabolic diseases (9). Previous studies have explored the characteristics of gut microbiota and its role in metabolizing key nutrients in insulin resistance. Carbohydrate metabolism by commensal organisms has been proposed to contribute up to 10% of total host energy, highlighting its potential role in the pathogenesis of prediabetes and obesity (10, 11).

Current methods for diagnosing, screening, and managing prediabetes remain limited due to an incomplete understanding of its pathophysiology. Genetic predisposition plays a role in diabetes risk, but environmental factors such as an unhealthy diet, a sedentary lifestyle, and smoking also contribute. Some diabetes-related environmental risk factors may exert their diabetogenic effects through alterations in the gut microbiota. The gut microbiota has the potential to influence adiposity and glucose metabolism, impacting human health and a variety of diseases. Altered composition and function of the gut microbiota have recently been implicated in the pathogenesis of type 2 diabetes (T2D) and other metabolic disorders (12).

Dysbiosis of the gut microbiota plays a significant role in metabolic disorders through several mechanisms, including increased gut permeability, low-grade endotoxemia, altered short-chain fatty acid (SCFA) production, and disruptions in branched-chain amino acid (BCAA) and bile acid metabolism. Changes in gut microbiota composition and function have been observed in individuals with prediabetes (13, 14). Although numerous studies have examined the relationship between gut microbiota and metabolic disorders, research specifically focusing on prediabetes remains scarce. Prediabetes represents a transitional phase toward T2D, yet the role of gut microbiota—particularly in inflammatory mechanisms and taxonomic abundance—has not been well clarified. Therefore, this review aims to systematically evaluate the correlation between gut microbiota, inflammation, and taxonomic abundance in prediabetes. Understanding these interactions may provide valuable insights into potential therapeutic strategies for preventing T2D. Additionally, diet is a major factor influencing gut microbiota composition, and an increasing number of diet-derived microbial metabolites have been linked to insulin secretion, insulin sensitivity, and the onset of T2D (15, 16).

## Materials and methods

### Protocol and registration

This systematic review was conducted following the Preferred Reporting Items for Systematic Reviews and Meta-Analyses (PRISMA) guidelines to ensure a structured and transparent approach. The protocol was registered in the International Prospective Register of Systematic Reviews (PROSPERO) under registration number CRD42025637369. A flow diagram will be provided to illustrate the study selection process.

### Search strategy and data extraction

We have defined the criteria for considering studies as follows: the PICO(S) framework. The population of interest comprised individuals with prediabetes, while the intervention focused on the composition, diversity, or alterations in gut microbiota. The comparison group included healthy individuals without prediabetes or those with a normal glycemic status. The primary outcomes assessed were inflammatory biomarkers, disease phenotypes related to prediabetes, and the abundance of specific gut microbiota taxa.

A comprehensive literature search was conducted using PubMed, ClinicalKey, ScienceDirect, Springer, and Scopus for studies published between 2015 and 2025. The search strategy incorporated a combination of Medical Subject Headings (MeSH) and free-text terms. The search terms included “prediabetes” OR “pre-diabetic” OR “impaired glucose tolerance” OR “impaired fasting glucose” OR “HbA1c” AND “gut microbiota” OR “gut microbiome” OR “intestinal microbiota” OR “intestinal microbiome” OR “microbiota composition” OR “microbiota diversity” OR “gut dysbiosis” OR “gut bacteria.” To improve specificity, the term “metabolic phenotype” was expanded into more precise concepts, including “glucose metabolism,” “lipid metabolism,” and “energy homeostasis.” Similarly, the terms “obesity,” “insulin resistance,” and “glucose” were further detailed as “body mass index (BMI),” “adiposity,” “hyperinsulinemia,” and “fasting plasma glucose.” Additionally, to capture relevant studies on inflammatory biomarkers, synonyms such as “cytokines,” “chemokines,” “pro-inflammatory markers,” “tumor necrosis factor (TNF),” and “interleukins” were incorporated into the search terms. To enhance search precision, search terms were adapted to meet the specific requirements of each database. A table summarizing the exact search terms, search dates, and databases will be included in the full report.

Data extraction was conducted independently by two reviewers and cross-checked by a third reviewer to ensure accuracy, using a standardized data extraction form to collect key study details. Extracted information included study characteristics such as author, year of publication, study design, and setting; participant characteristics including age, sex, BMI, and diagnostic criteria for prediabetes; microbiota assessment covering specific gut microbiota taxa is analyzed and methods of assessment; inflammatory biomarkers such as cytokines, chemokines, and other inflammation markers were measured; and outcome measures including effect sizes, confidence intervals, and intervention details if applicable. Any disagreements during the extraction process were resolved through discussion or consultation with a fourth reviewer.

### Eligibility criteria

The study designs considered for inclusion were observational studies, including cohort, case–control, and cross-sectional studies, as well as intervention studies. Studies were included if they assessed the relationship between gut microbiota and prediabetes, reported specific gut microbiota taxa and their role in prediabetes, and measured inflammatory biomarkers related to gut microbiota and metabolic changes. Additionally, studies were required to define prediabetes using standardized criteria such as HbA1c levels, fasting glucose levels, or the oral glucose tolerance test (OGTT). Only studies published in English were considered. Exclusion criteria included reviews, editorials, case reports, and studies without primary data.

### Risk of bias assessment

Accordingly, we extracted information from our chosen articles, which included author and year of publication, study design and settings, and findings. The results were summed up and introduced using a table. Quality assessment was conducted collaboratively by three reviewers until consensus was reached. The Newcastle–Ottawa Scale (NOS) and the Joanna Briggs Institute (JBI) tool were used to assess methodological quality.

## Results

### Study selection

**Figure 1:**
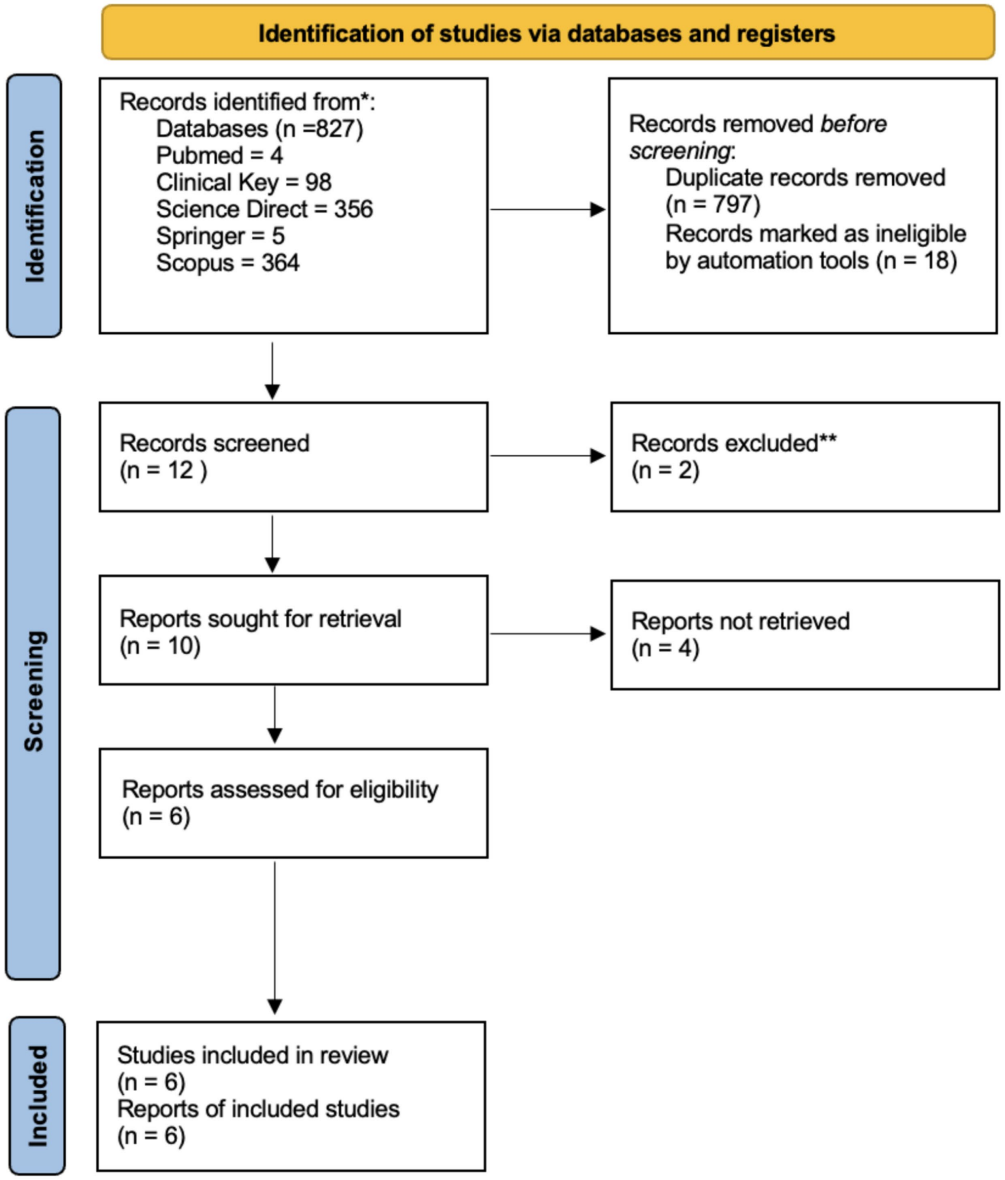
PRISMA flow diagram for the new systematic reviews, which included searches of databases and registers only.

Our search results identified 827 publications. After screening by title and abstract, a total of 6 studies matched the eligibility criteria. After reading the full text, 6 articles were decided to be included in the study. We used the PRISMA flow diagram for searching and extracting data and presented it on the PRISMA flow diagram (Figure 1).

### Quality assessment and data synthesis

The selected articles were then assessed for quality and risk of bias using the Newcastle–Ottawa Scale (NOS) and the Joanna Briggs Institute (JBI) tool (Supplementary Table S1). Cross-sectional and case–control studies use the JBI Critical Appraisal Checklist. While, Cohort studies use Newcastle–Ottawa Scale (NOS) Critical Appraisal Checklist. Any discrepancies were addressed until a solution was found by three reviewers.

### Study characteristics

The reviewed studies demonstrate significant associations between gut microbiota composition and metabolic conditions, including prediabetes and type 2 diabetes (T2D). The study by Wu et al. (14) indicated that diabetic individuals present differences in their gut microbiota profile from that of prediabetic individuals. T2D individuals with reduced butyrate-producing bacteria were said to correlate these changes with the insulin-resistance condition. Similarly, Pinna et al. (12), while working with examination, reported a particular operational taxonomic unit (OTU) occurring in normoglycemia—for example, described by Prevotella 9—and another OTU occurring in prediabetes—indicative of, say, Streptococcus—with peculiar inflammation-related patterns of microbiota in an Indian population. Increases in prediabetes were identified to be bacterial taxa such as *Dorea* and *Ruminococcus* by Allin et al. Meanwhile, *Akkermansia muciniphila* was notably reduced. Higher insulin-resistance association was found by Takeuchi et al. (11) for bacterial families such as Lachnospiraceae, whereas more fecal carbohydrate levels were again associated with inflammation. Exercise and weight loss, though, added to these similar microbiota effects over diet-based interventions, as pointed out by Beals et al. (8). Finally, altered microbiota, especially lower short-chain fatty acid producers, is demonstrated in T2D but not in prediabetes, suggesting that these changes occur at later disease stages. All these findings indicate the importance of gut microbiota in metabolic dysregulation, inflammation, and possible therapeutic strategies for the management of prediabetes and T2D (Table 1).

**Table 1:** Characteristics of the included articles.

Author	Study design	Population	Respondents’ Characteristics
Wu H. et al. (14)	Cross-sectional	The study included men and women aged 50–64 years who were randomly recruited from the census register in the Gothenburg area, Sweden. They profiled the gut microbiota in a discovery (*n* = 1,011) and validation (*n* = 484) cohort comprising Swedish subjects naive to diabetes treatment and grouped by glycemic status. Diagnosis based on glucose fasting and a 2 h oral glucose tolerance test (OGTT) of capillary plasma glucose levels and is divided into subgroups using the 1999 World Health Organization (Geneva) criteria.	*n* = 226 for low-risk normoglycemic participants. *n* = 297 for high-risk normoglycemic participants. *n* = 189 for IFG participants (impaired fasting glucose). *n* = 75 for CGI participants (combined glucose intolerance). *n* = 46 for T2D participants.
Pinna K. N. et al. (12)	Cross-Sectional	A total of 259 Danish volunteers—138 normoglycemic control and 121 with prediabetes—were recruited. All Danish subjects were of white European ethnicity, aged 35 to 74 years, with a body mass index of 20 to 40 kg/m^2^. Diagnosis of normal glucose tolerance and impaired glucose tolerance was based on the results of a standard oral glucose tolerance test (OGTT), performed using an 82.5 g oral glucose load (equivalent to 75 g of anhydrous glucose). Individuals with fasting plasma glucose of 6.1 to 6.9 mmol/L or glycated hemoglobin A1c of 5.7 to 6.4% (39 to 47 mmol/mol) are prediabetics.	*n* = 259 Danish volunteers, 138 normoglycemic controls and 121 with prediabetes. *n* = 278 Indian individuals, 137 with normal glucose tolerance and 141 with prediabetes.
Allin K. H. et al. (8)	Case-Control	The study included 134 individuals with prediabetes and 134 individuals with normal glucose regulation. Diagnosis of prediabetes, according to WHO criteria, was defined as a fasting plasma glucose level of 6.1–7.0 mmoL/L or an HbA1c level of 42–48 mmol/mol (6.0–6.5%). Normal glucose regulation was defined according to the more stringent ADA criteria: fasting plasma glucose <5.6 mmoL/L and HbA1c < 39 mmol/mol (5.7%)	*n* = 134 Danish adults with prediabetes, overweight, insulin resistance, dyslipidemia, and low-grade inflammation. *n* = 134 age- and sex-matched individuals with normal glucose regulation.
Takeuchi T. et al. (11)	Cross Sectional	The study included a total of 306 Japanese people (71% male) without diabetes, not under any treatment for intestinal diseases, and with no prior use of antibiotics for 2 weeks, aged from 20 to 75 years, who were recruited during annual health check-ups. Diagnosis of prediabetes according to glycated hemoglobin (HbA1c) was 5.8% (5.5–6.1%).	*n* = 306 Japanese adults without diabetes, not under any treatment for intestinal diseases, and with no prior use of antibiotics for 2 weeks. *n* = 112 had normal metabolic profiles. *n* = 100 were obese. *n* = 101 were prediabetes.
Beals J. W. et al. (8)	Experimental study	The study included a total of 21 adults with obesity and prediabetes who participated in one of two interventions (Diet-ONLY, *n* = 11; diet + exercise, *n* = 10), with BMI 30.0–49.9 kg/m^2^. Prediabetes was described as HbA1c 5.7–6.4% or fasting plasma glucose 100–125 mg/dL, or 2-h OGTT plasma glucose concentration 140–199 mg/dL.	*n* = 21 adults with obesity and prediabetes participated. *n* = 11 undergo Diet-ONLY intervention. *n* = 10 undergo Diet+Exercise intervention.
Maskarinec G. et al. (16)	Cross-sectional	The study included a total of 1702 individuals from five ethnic groups who completed clinical visits, questionnaires, and stool and blood collection. Diagnosis of prediabetes is based on fasting glucose <125 mg/dL and 100–125 mg/dL.	*n* = 1702 participants from five ethnic groups (white, African American, Native Hawaiian, Japanese American, and Latino) aged 58–74 years. *n* = 735 were normoglycemic. *n* = 506 were prediabetes. *n* = 154 were undiagnosed T2D. *n* = 307 were T2D.

## Discussion

Diabetes is a chronic disease caused by an inherited or acquired deficiency in insulin production by the pancreas or the body’s inability to use the insulin it produces adequately (17). The natural history of T2D includes a stage of prediabetes, in which blood glucose levels are higher than normal but not high enough for a diabetes diagnosis. Prevention of the disease is possible at this stage. Several studies with limited sample sizes have reported a possible association between gut microbiome composition and prediabetes (12). Studies have shown diverse associations between the gut microbiome and diabetes in European, American, and Chinese cohorts. However, the specific bacteria associated with diabetes vary across populations (18). In the population-based cross-sectional study by Hao Wu et al. (14), significant changes in the gut microbiota were observed at both compositional and functional levels in diabetes treatment-naive T2D individuals in the discovery cohort. These findings were confirmed in the validation cohort. Importantly, changes in the gut microbiota were already present in individuals with prediabetes but were more pronounced in those with CGI and strongly associated with insulin resistance. This supports the hypothesis that the gut microbiota is a modifiable determinant of T2D closely linked to dietary factors. Future studies should examine whether restoring altered microbial function, such as butyrate production, in individuals with IGT and CGI might help prevent or delay the progression to T2D (14).

Observations from the study by Pinna et al. (12), combined with phenotypic data and inflammatory biomarker levels, suggest that the gut microbiome’s role in the pathophysiology of prediabetes in Indian subjects differs from that in European populations. While chronic systemic inflammation is characteristic of the general Indian population, the observed anti-inflammatory and protective effects induced by factors in the Indian gut microbiome appear crucial in determining gut health and modulating diabetes onset and progression. In the study by Allin et al. (8), individuals with prediabetes showed higher fasting plasma glucose, insulin, C-peptide, triacylglycerol, hsCRP, HbA1c, HOMA-IR, BMI, and waist circumference compared to normoglycemic individuals. Although genetic differences and disease phenotype expression exist, microbial composition changes in individuals with prediabetes appear common across diverse pathological phenotypes characterized by low-grade inflammation. The most significant microbial alteration is the depletion of *Clostridium* and *Akkermansia muciniphila* (1).

Most studies (Table 2) reveal reduced alpha diversity with the depletion of butyrate-producing bacteria, such as *Faecalibacterium prausnitzii* and *Roseburia*, compared to normoglycemic controls. Furthermore, prediabetes is associated with a significant enrichment of *Escherichia/Shigella, Streptococcus, Prevotella-2, Vibrio,* dan *Alloprevotella*, with some showing more than a 4-fold increase. These microbial changes correlate with systemic inflammation, elevated IL-6, TNF-*α*, and CRP, as well as insulin resistance (HOMA-IR). Geographic and dietary factors have influenced the microbiota and shaped unique microbiome profiles related to glucose intolerance. This has resulted in an altered gut microbiota characterized by dysbiosis, low-grade inflammation, and reduced SCFA production, playing a major role in prediabetes pathogenesis.

**Table 2:** Summary of gut microbiota diversity in prediabetes and its association with inflammation and taxonomic abundance.

Author	Inflammation & insulin resistance	Altered gut microbiota composition	Reduction in butyrate-producing bacteria	Specific taxa changes
Wu, H et al.	Insulin resistance was strongly associated with microbial variations (*p* < 0.05).	Significant microbiota shifts occur in prediabetes and T2D but not in impaired fasting glucose. Principal coordinate analysis showed marked differences (p_adj < 0.05) in microbiota between IGT, CGI, and T2D vs. normoglycemia.	*Faecalibacterium* were *Roseburia reduced* in prediabetes (*p* < 0.05). Functional potential for butyrate production was significantly lower.	**Increased:** *Streptococcus, Escherichia/Shigella, Prevotella-2,* and *Megasphaera* (p_adj < 0.05). Differential abundance testing (negative binomial model) showed significant shifts.
Pinna, KN et al.	Higher inflammatory markers (IL-6, TNF-*α*) in prediabetes (*p* < 0.05). Dysbiosis is associated with inflammation.	Prediabetes was associated with Klebsiella, Escherichia/Shigella, and Prevotella-2 (*p* < 0.05). 144 OTUs were significantly altered *(Streptococcus, Escherichia/Shigella).*	Significant decline in butyrate-producing bacteria (*p* < 0.05).	**Increased:** *Streptococcus, Vibrio, Alloprevotella* (≥4-fold in prediabetes, p_adj < 0.05).
Takeuchi, T et al.	Insulin resistance (HOMA-IR ≥ 2.5) was associated with gut microbiota changes (*p* < 0.05). Cytokines (IL-10, adiponectin, and leptin) mediate the gut microbiota’s effects on insulin resistance.	36 OTUs significantly differed between prediabetes and normoglycemia (p_adj < 0.01). Fecal carbohydrates (fructose, glucose, and galactose) positively correlated with BMI and insulin resistance (*p* < 0.05).	*Faecalibacterium prausnitzii* was reduced by 30% in prediabetes (*p* < 0.01).	**Increased:** *Dorea, Ruminococcus, Sutterella, Streptococcus* (log2 fold change 0.51–0.92, p_adj < 0.05). **Decreased:** *Clostridium* (log2 fold change −0.64, p_adj = 0.0497), *Akkermansia muciniphila* (log2 fold change −1.74, p_adj = 2 × 10^−3^).
Allin, KH et al.	Low-grade inflammation and insulin resistance were linked to gut microbiota changes (*p* < 0.05).	Prediabetes microbiota alterations resemble metabolic disorders. Higher *Lachnospiraceae (Blautia, Dorea)* and *Actinobacteria* were associated with insulin resistance, while *Bacteroidales (Bacteroides, Parabacteroides, Alistipes)* and *Faecalibacterium* were protective (p_adj < 0.05).	Inconsistent butyrate-producer reduction: some *Faecalibacterium prausnitzii* strains increased while others decreased.	**Increased:** *Dorea, Ruminococcus, Sutterella, Streptococcus* (p_adj < 0.05). Decreased: *Clostridium, Akkermansia muciniphila* (log2 fold change −1.65, p_adj = 4 × 10^−4^).
Beals, JW et al.	Increased gut permeability led to higher CRP (*p* < 0.01).	Prediabetes individuals had lower microbiota diversity (*p* < 0.05). Dietary interventions significantly altered the microbiota composition. Exercise + diet doubled insulin sensitivity vs. diet alone, linked to gut microbiota changes (*p* < 0.05).	*Faecalibacterium prausnitzii* was significantly lower in prediabetes (*p* < 0.01).	Beta diversity changes were significant from baseline (T0) to 1 month (T1) (PERMANOVA, *p* < 0.05).
Maskarinec, G et al.	Higher bacterial translocation (LPS) associated with systemic inflammation (CRP, LBP, *p* < 0.05).	Dysbiosis in prediabetes and T2D. *Actinobacteria* and *Firmicutes* were lower in T2D; *Proteobacteria* were more abundant. Gut microbiota alterations are correlated with metabolic inflammation (*p* < 0.05).	Significant reduction in SCFA-producing bacteria, including Roseburia and *Faecalibacterium prausnitzii* in prediabetes (*p* < 0.05).	Shift toward pro-inflammatory taxa in T2D, favoring *Proteobacteria* over beneficial *Firmicutes* (*p* < 0.05).

Metagenomic studies show that approximately 90% of gut bacterial species in adults belong to *Bacteroidetes* and *Firmicutes*. *Actinobacteria*, *Proteobacteria*, and *Verrucomicrobia* are present in smaller proportions (18). The composition of the microbiome is influenced by many factors, and while the current study set out to find associations of microbial taxa with glycemic status, several measured covariates, including the subject’s physical/biochemical parameters as well as markers of inflammation, could influence the observed microbiome state (12). In the experimental study by Takeuchi et al. (11), individuals with abundant gut microbiota showed a higher proportion of insulin resistance. *Escherichia/Shigella, Streptococcus, and Prevotella-2* showed a more than 4-fold increase in abundance and are known to ferment dietary fiber and produce metabolites such as SCFAs (19). Insulin resistance is also linked to elevated SCFA levels and has an association with its function in gluconeogenesis (20, 21). This study also showed that the end products of carbohydrate degradation, such as monosaccharides, which are readily absorbed and used by humans, are particularly increased in the feces of individuals with insulin resistance and metabolic syndrome. The abundant accumulation of monosaccharides has the potential to promote ectopic lipid accumulation and also trigger immune cells, resulting in low-grade inflammation and insulin resistance. This study also showed that IL-10 has the most prominent associations with fecal carbohydrates and shows its paradoxical effect to facilitate IR. Fructose is recognized as a key contributor to inflammation and insulin resistance due to its role in lipid accumulation, while galactose has a role in activating immune cells in energy metabolism (21–23).

Beals et al. (8) demonstrated that significant alterations in gut microbiota composition are closely linked to conditions such as obesity, metabolic syndrome, and diabetes. Unlike earlier studies that primarily focused on the relationship between gut microbiota and prediabetes, this study explored a novel approach by assessing whether the effects of administering or transplanting gut microbiota from healthy, lean individuals into participants with obesity and metabolic syndrome could enhance insulin sensitivity. Additionally, this study included an analysis of fecal samples collected at baseline. It found that a plant-based diet led to a substantial change in gut microbiota beta diversity, indicating a notable change in microbial composition. However, this study was unable to determine whether these changes in gut microbiota directly contributed to the insulin sensitivity improvements observed in the participants or were simply related to changes in dietary composition, which may not have independently influenced insulin action.

Finally, a cross-sectional study by Maskarinec et al. (16) showed quite different results compared to the studies that had been presented previously. A decrease in SCFA-producing bacteria, such as those producing butyrate, and a rise in Gram-negative, endotoxin-producing bacteria are observed in T2D cases but not in prediabetes cases. Prediabetes values were closer to normoglycemic cases than T2D. This shift might reflect an unfavorable microbiome composition in T2D. These studies are limited by their cross-sectional design, making it difficult to establish causality. Additionally, selection bias may influence the microbiota variations observed in different populations.

Specific genera within *Firmicutes* and *Proteobacteria* were found to be significantly more abundant in T2D cases. This indicates that certain bacterial groups may flourish in the altered gut environment associated with T2D. SCFAs have anti-inflammatory and gut barrier-supporting roles. SCFAs, especially butyrate, have been shown to help maintain gut physiologic hypoxia through several mechanisms to maintain tight junctions and gut epithelial integrity (24). Disruption of these pathways—reduced SCFA production, increased gut hypoxia, impaired tight junction integrity, and changes in microbial diversity—can negatively impact host health, suggesting that their reduction may exacerbate gut and systemic inflammation. LPS triggers Toll-like receptor 4 (TLR4) activation, leading to NF-κB pathway stimulation and increased pro-inflammatory cytokine production, contributing to insulin resistance. These alterations may promote systemic inflammation, reduce energy homeostasis, impair insulin sensitivity, and increase the risk of developing or exacerbating T2D (25). Epidemiologic evidence links the gut microbiome to T2D, with reduced butyrate-producing bacteria from the Firmicutes phylum commonly observed in T2D patients and more likely to have a higher presence of opportunistic pathogens (26, 27).

Metabolic endotoxemia refers to persistent, low-grade systemic inflammation linked to obesity and metabolic diseases, including T2D (28). Increased abundance of endotoxin-producing bacteria, particularly in the Escherichia/Shigella group (e.g., *Escherichia coli*) and other Proteobacteria, has been reported in T2D patients (28, 29). These bacteria contribute to endotoxin production, which is implicated in metabolic dysfunction. Endotoxins can trigger systemic inflammation via bacterial translocation across the gut barrier, a process implicated in metabolic dysregulation. The microbiome changes support the hypothesis that unfavorable bacterial patterns may contribute to T2D by promoting chronic low-grade inflammation (30) (Table 3).

**Table 3:** Some potentially harmful and potentially beneficial gut microbiota bacterial (31–35).

Bacteria	Associated physiologic changes	Inflammation marker (increased or decreased)	Associated disease states
*Escherichia/Shigella*	Increased gut dysbiosis, potential endotoxin production	↑ IL-6, TNF-α	Prediabetes, Diabetes
*Klebsiella*	Increased gut inflammation, disrupted intestinal barrier	↑ CRP, LPS-binding protein	Prediabetes
*Prevotella-2*	Dysregulated immune response, increased intestinal inflammation	↑ IL-6, TNF-α	Prediabetes
*Streptococcus*	Altered gut microbiota composition, potential gut inflammation	↑ IL-6, TNF-α	Prediabetes
*Megasphaera*	Increased gut dysbiosis, potential association with insulin resistance	↑ IL-6, TNF-α	Prediabetes
*Faecalibacterium prausnitzii*	Reduced butyrate production, disrupted gut health	↓ IL-10, adiponectin	Prediabetes, Diabetes
*Roseburia*	Reduced short-chain fatty acid (SCFA) production, impaired gut health	↓ IL-10, adiponectin	Prediabetes
*Blautia*	Reduced butyrate production, impaired gut microbiota balance	↓ IL-10, ↑ C-reactive protein	Prediabetes
*Dorea*	Altered gut microbial composition, impaired metabolic health	↑ CRP, TNF-α	Prediabetes
*Akkermansia muciniphila*	Improved gut health, enhanced mucin layer and barrier function	↓ IL-6, increased adiponectin	Prediabetes
*Faecalibacterium (Other strains)*	Decreased in abundance in prediabetes, loss of anti-inflammatory effects	↑ CRP, TNF-α	Prediabetes
*Clostridium*	Decreased abundance, associated with lower glucose tolerance	↑ IL-6, TNF-α	Prediabetes
*Firmicutes*	Increased in T2D, altered balance of gut microbiota	↑ IL-6, CRP	Type 2 Diabetes
*Proteobacteria*	Increased endotoxin production, dysbiosis	↑ LPS-binding protein, CRP	Type 2 Diabetes
*Actinobacteria*	Reduced abundance, impaired immune regulation	↓ IL-10	Prediabetes Diabetes
*Alistipes onderdonkii*	Increased gut dysbiosis, loss of beneficial bacteria	↑ IL-6	Type 2 Diabetes

Notably, these patterns were not observed in prediabetic participants, indicating their role may be specific to advanced metabolic dysfunction. But these results emphasize the gut microbiome’s involvement in T2D, providing insights for potential microbiome-targeted interventions to manage or prevent the disease. Specific microbiome signatures, such as the depletion of butyrate-producing *Faecalibacterium prausnitzii* and *Roseburia* and increased levels of pro-inflammatory taxa like *Escherichia/Shigella* and *Prevotella-2,* may identify at-risk individuals and forecast disease progression. Additional gut-derived metabolites that provide insight into host-microbiome interactions affecting insulin sensitivity and systemic inflammation include SCFAs and endotoxins. Apart from diagnosis, microbiome-targeted interventions, especially dietary changes, probiotics, and fecal microbiota transplantation (FMT), have some promise in restoring microbial imbalance and restricting metabolic health. Future research that links microbiome profiling with genetics, metabolism, and inflammatory markers may use machine learning and multi-omics approaches to improve predictions and personalize treatment strategies for metabolic disorders.

## Conclusion

The gut microbiota present aberrancy in prediabetes. In prediabetes, gut microbiota dysbiosis, which consists of reduced butyrate-producing bacteria, contributes to both systemic inflammation and insulin resistance. Restore the microbiota balance, paving the way for microbiome-based treatments to prevent and treat prediabetes.

## Data Availability

The original contributions presented in the study are included in the article/Supplementary material, further inquiries can be directed to the corresponding author.
