# Supplementary material for: Implication of taxonomic abundance of gut microbiota in prediabetes: a systematic review
Source: Front Nutr. 2025 Apr 16;12:1577528. doi: 10.3389/fnut.2025.1577528 (PMC12042084; doi:10.3389/fnut.2025.1577528)
Supplement: Supplementary file 1 [file Table_1.docx]

**Supplementary**

Table S1. The Newcastle-Ottawa Scale (NOS) and Joanna Briggs Institute (JBI) for Assessing

The Quality of Studies

| **Author** | **Year** | **Study Design** | **NOS**  **(0-9)** | **JBI** | **Selection** | **Comparability** | **Outcome** | **Overall Risk of Bias** |
| --- | --- | --- | --- | --- | --- | --- | --- | --- |
| **Wu, H et al.** | 2020 | Cross-sectional | 7 |  | Good | Good | Good | Low (NOS) |
| **Pinna, KN et al.** | 2021 | Experimental |  | Low Risk | N/A | N/A | N/A | Low (JBI) |
| **Allin, KH et al.** | 2018 | Case Control | 8 |  | Good | Good | Good | Low (NOS) |
| **Takeuchi, T et al.** | 2023 | Cross-sectional | 7 |  | Good | Good | Good | Low (NOS) |
| **Beals JW et al.** | 2023 | Experimental |  | Low Risk | N/A | N/A | N/A | Low (JBI) |
| **Maskarinec G et al.** | 2021 | Cross-sectional | 7 |  | Good | Good | Good | Low (NOS) |
